# Association Between Physical Activity, Sedentary Behavior and Breast Cancer Risk Among Moroccan Women: A Multicenter Case–Control Study

**DOI:** 10.3390/epidemiologia7010022

**Published:** 2026-02-03

**Authors:** Siham Mrah, Najoua Lamchabbek, Mounia Amzerin, Najia Mane, Nawfel Mellas, Karima Bendahou, Chaimaa Elattabi, Saber Boutayeb, Lahcen Belyamani, Elodie Faure, Inge Huybrechts, Adil Najdi, Fatima Zahra El M’rabet, Mohamed Khalis

**Affiliations:** 1 Laboratory Research of Cancer and Chronic Diseases, Faculty of Medicine and Pharmacy of Tangier, Abdelmalek Essaadi University, Tetouan 93000, Morocco; m.amzerin@uae.ac.ma (M.A.); a.najdi@uae.ac.ma (A.N.); fazoumed@gmail.com (F.Z.E.M.); 2 Department of Public Health and Clinical Research, Mohammed VI Center for Research and Innovation, Rabat 10112, Morocco; nlamchabbek@um6ss.ma (N.L.); celattabi@um6ss.ma (C.E.); sboutayeb@cm6.ma (S.B.); belyamani@gmail.com (L.B.); mkhalis@um6ss.ma (M.K.); 3 Mohammed VI International School of Public Health, Mohammed VI University of Sciences and Health, Casablanca 82403, Morocco; 4 Laboratory of Epidemiology and Research in Health Sciences, Faculty of Medicine, Pharmacy and Dental Medicine, Sidi Mohamed Ben Abdallah University, Fez 30070, Morocco; najiasaidimane@gmail.com; 5 Department of Oncology, Hassan II University Hospital of Fez, Fez 30050, Morocco; mellasnawfel@yahoo.fr; 6 Department of Epidemiology, Faculty of Medicine and Pharmacy of Casablanca, Hassan II University of Casablanca, Casablanca 9154, Morocco; bendahou.karima@gmail.com; 7 Department of Medical Oncology, National Institute of Oncology, Rabat 6213, Morocco; 8 CESP, Inserm U1018, Université Paris-Saclay, UVSQ, Gustave Roussy, 94805 Villejuif, France; elodie.faure@gustaveroussy.fr; 9 International Agency for Research on Cancer, World Health Organization, 69366 Lyon, France; huybrechtsi@iarc.who.int; 10 French Network for Nutrition and Cancer Research (Nacre Network), 78350 Jouy-en-Josas, France; 11 Higher Institute of Nursing Professions and Health Techniques, Rabat, Ministry of Health and Social Protection, Rabat 10000, Morocco

**Keywords:** physical activity, breast cancer, case–control study, sedentary behavior, occupational activity

## Abstract

Purpose: Breast cancer (BC) incidence has been increasing rapidly in North Africa, including Morocco, yet evidence regarding modifiable lifestyle factors remains limited. This study aimed to assess the associations between physical activity, sedentary behavior, daily work habits, and BC risk among Moroccan women, addressing an important gap in regional data. Methods: We conducted a case–control study between 2019 and 2023, including 1400 histologically confirmed incident BC cases and 1400 matched controls. Physical activity was assessed across the lifespan, considering type, intensity, and duration. Associations with BC risk were estimated using adjusted odds ratios (aORs) and 95% confidence intervals (CIs). Results: Moderate physical activity was inversely associated with BC risk, showing a clear dose–response relationship. Compared with the lowest physical activity level, the highest quartile showed significantly lower odds of BC (aOR = 0.37 (95% CI: 0.29–0.47). Vigorous physical activity during young adulthood and mid-adulthood was similarly linked to reduced risk. Active daily habits, such as walking and regular stair climbing, were associated with lower odds, whereas frequent occupational fatigue and sweating were linked to increased risk. Conclusions: Our findings highlight a significant inverse association between physical activity and BC risk among Moroccan women. Notably, moderate PA and active daily habits like brisk walking are linked to lower odds of the disease. While these findings support the role of physical activity as an important factor associated with breast cancer prevention, the retrospective design of the study limits causal inference.

## 1. Introduction

Breast cancer (BC) represents a serious global public health burden, with 2.3 million new cases reported in 2022 and approximately 666,000 deaths reported in the world [1]. While incidence rates remain higher in high-income countries [2], an alarming upward trend in mortality is emerging in low-income regions. In Africa, BC has become the leading cause of cancer death [3,4,5]. This is particularly critical in Morocco, where BC is the most common cancer diagnosis among women, accounting for 38.1% of all female cancers [6].

The identification of the risk factors associated with BC is essential to primary prevention of the disease [7,8,9]. While some risk factors such as genetic predisposition remain non-modifiable, it is estimated that 25–30% of BC cases could be prevented by healthy life style including regular physical activity [10]. Interest in the link between PA and cancer prevention dates back to the mid-20th century [11], as physical inactivity has become a critical global health issue associated with millions of annual deaths [12,13].

Numerous studies have reported a dose–response effect where BC risk decreases as PA levels increase [14,15,16,17]. Systematic reviews and meta-analyses of large epidemiological cohorts consistently support the preventive role of physical activity in breast cancer etiology [15,16]. These studies highlight a definitive dose–response relationship, indicating that moderate-to-vigorous physical activity provides a 10–25% reduction in risk [17,18]. This benefit appears universal, applying to both pre- and post-menopausal women across different molecular subtypes of the disease [16,19]. These protective effects are mediated by complex biological mechanisms, including the regulation of sex hormones (notably estradiol), improved immune function, and reduced chronic inflammation or insulin resistance [15,20]. The World Health Organization (WHO) emphasizes PA as a cornerstone of prevention, which, when combined with healthy weight management and limited alcohol/tobacco use, can reduce BC risk by up to 30% [21]. However, sedentary behavior has emerged as an independent risk factor for mortality and BC [21,22]. Although its association with BC risk through obesity-related pathways has shown some inconsistency in previous studies [23], it remains a critical factor to evaluate alongside PA.

While recreational physical activity is a well-known protective factor, the impact of occupational physical demands remains less clear, particularly in transitioning societies. In Morocco, many women are exposed to significant physical strain through daily work habits, including heavy lifting and labor-intensive tasks. Evaluating proxies of work intensity, such as occupational sweating and fatigue, is essential to capture the full spectrum of physical activity and its potential biological impact on BC risk in this specific population.

In this context, the present study aims to estimate the associations between physical activity levels, sedentary behavior patterns, occupational routines and BC risk among Moroccan women. By identifying the modifiable determinants of this disease, this research aims to contribute to prevention efforts and the improvement of public health, both in Morocco and around the world. Despite the rising incidence of BC in North Africa, data regarding the protective role of specific physical activity domains remain sparse. This study addresses this gap by examining life-course activity and occupational habits in Moroccan women. We hypothesized that regular physical activity and active daily routines are significantly associated with reduced odds of BC, while prolonged sedentary behavior is linked to increased risk.

## 2. Materials and Methods

### 2.1. Study Design and Setting

This large-scale case–control study, part of the BREAST MOROCCO study, included a total of 1400 BC cases and 1400 controls. Participants were recruited from various hospitals centers across Morocco between December 2019 and August 2023. The research was conducted at multiple sites, including Ibn Rochd University Hospital in Casablanca, Hassan II University Hospital in Fez, Al Hoceima Oncology Centre, Sheikh Zayed Al Nahyan Oncology Hospital in Tangier, and Al Hassani Provincial Hospital in Nador. Sample Size Calculation: The sample size was determined to detect an odds ratio (OR) of 0.75 for physical activity with 80% power and a 5% significance level, assuming a 20% prevalence of high physical activity among controls. This required a minimum of 1254 cases and 1254 controls. Our final recruitment of 1400 pairs (*n* = 2800) ensures robust statistical power for the conducted multivariable adjustments.

### 2.2. Study Population

BC cases were defined as women newly diagnosed with histologically confirmed BC, either in situ or invasive, who had not received any cancer related treatment (e.g., chemotherapy, radiotherapy, hormonal therapy) and had no prior history of any other cancer.

Control participants were healthy women with no history of cancer, randomly selected from individuals visiting or accompanying patients with non-cancer-related conditions at the same hospitals where cases were recruited. Controls were individually matched to cases based on age (±5 years) and home of residence (urban or rural). While hospital-based controls may introduce potential selection bias, they were recruited from the same geographical areas as cases to ensure comparability in terms of environmental exposures and socio-economic background.

All participants provided voluntary informed consent and were not pregnant or breastfeeding at the time of data collection. The participation rates were with 98.2% for cases and 93.8% for controls.

### 2.3. Ethical Consideration

The study protocol was approved by the Research Ethics Committee of the Faculty of Medicine and Pharmacy of Casablanca (N^o^.09/16, approved on 3 March 2016). The present analysis is based on the same ethical approval, as no changes were made to the study protocol or data collection procedures All participants provided written informed consent before participation, after being informed about the study’s objectives, confidentiality measures, and their right to withdraw at any time.

### 2.4. Data Collection

Data were collected through standardized face-to-face interviews conducted by six trained interviewers, using two questionnaires: a general questionnaire and a validated food frequency questionnaire (FFQ), as described previously [24,25].

The comprehensive questionnaire covered a range of socio-demographic characteristics (age, marital status, education, and wealth score based on household assets), occupational history, family history of cancer, menstrual and reproductive factors (age at menarche, age at first full-term pregnancy, parity, menopausal status, age at menopause, breastfeeding history), hormone use, including oral contraceptives and hormone replacement therapy, smoking history, alcohol consumption and physical activity. In addition, anthropometric measurements (height, weight, waist and hip circumference) were recorded. Dietary intake and daily energy consumption (kcal/day) were assessed using the Moroccan-validated 255-item FFQ [26].

### 2.5. Physical Activity Assessment

To comprehensively assess physical activity levels, a structured questionnaire was administered, designed to capture participants’ engagement across various domains of physical activity, including occupational, recreational, and household activities. Participants were systematically requested to report the duration of each activity category, classified by intensity as light, moderate, and vigorous, for every day of a typical week over the preceding 12 months. This detailed recall period aimed to provide a representative overview of their habitual physical activity patterns.

Although self-reported physical activity is subject to potential recall bias, the use of standardized, face-to-face interviews conducted by trained staff helped to minimize reporting errors and improve the reliability of the habitual activity data. To ensure accurate reporting and consistent interpretation, trained interviewers provided clear examples for each intensity level.

Additionally, the questionnaire included other aspects of physical activity and sedentary behavior allowing for a detailed understanding of participants’ physical activity profiles across various intensities, domains, and life stage:

- Overall activity level: Participants categorized their overall activity level over the past year into five categories: Sedentary, slightly active, moderately active, and highly active.
- High-intensity physical activity: Participants were asked if they engaged in high-intensity physical activity at least once per week, sufficient to increase breathing, heart rate, or cause sweating. Further, vigorous physical activity was specifically assessed for different age periods: between 6 and 11 years, 12 and 18 years, 19 and 25 years, 26 and 35 years, and during the past 5 years.
- Daily work habit or routine activities: For work-related activities, participants reported the frequency (Never, Rarely, Sometimes, Often and Very Often) of tasks such as sitting, standing, walking, and lifting heavy objects. Additional questions assessed the frequency of perceived fatigue after work and sweating during tasks.
- Commuting and daily walking: The questionnaire inquired about walking for commuting to work or school, including walking pace (slowly, normal pace, briskly) and duration (0–15 min, 16–30 min, 31–60 min, 1–2 h).
- Stair Climbing: Habits and frequency of stair climbing were assessed, with options for daily frequency (Once, Twice, Three times, Four times, more than five times per day).
- Sedentary behavior: Daily sitting time was quantified for weekdays and weekends (in hours per day), encompassing activities like watching television, computer use, and socializing while seated. This was used to categorize sedentary behavior as either less than 4 h/day or 4 h/day or more [27].

### 2.6. Statistical Analysis

Descriptive statistics were used to compare the characteristics of cases and controls. Categorical variables were expressed as frequencies and percentages, while continuous variables were presented as means with standard deviations. The characteristics of cases and controls were compared using *t*-tests for continuous variables and chi-square tests for categorical variables.

To assess the associations between BC risk and various factors, including physical activity and sedentary behavior, multivariable logistic regression models were applied. These models were adjusted for a comprehensive set of potential confounders, selected based on their established associations with BC risk in the literature and their potential to influence the observed relationships to minimize residual confounding (Supplementary Table S1). Variables were selected for inclusion in the final multivariate model based on a bivariate significance threshold (*p* < 0.20) and their established theoretical importance as confounding factors in breast cancer epidemiology (e.g., age, BMI, and family history). The categorization of continuous variables, such as moderate physical activity duration (in quartiles) and daily walking duration (in clinically relevant thresholds, e.g., 16–30 min), was performed to facilitate the assessment of potential dose–response relationships (*p*-trend) while ensuring adequate sample size per category for statistical stability. The adjustment variables included: age at menarche (continuous, in years), daily energy intake (continuous, in kcal/day), wealth score, education level (categorized as illiterate, elementary/Koranic, secondary, high school/technical), occupation (categorized as housewife, currently employed, previously employed), history of oral contraceptive use (yes/no), age at first full-term pregnancy (categorized as nulliparous, <22 years, ≥22 years), cumulative breastfeeding duration (categorized as never, >0–<24 months, ≥24 months, nulliparous), age at menopause (categorized as premenopausal, <50 years, ≥50 years), family history of BC (yes/no) body mass index (categorized as <25 kg/m^2^, 25–29 kg/m^2^, ≥30 kg/m^2^).

For ordinal variables, such as physical activity duration quartiles, self-reported physical activity level, and various daily work habit frequencies, *p*-trends were calculated. High-intensity physical activity was stratified by life stage (6–11 years, 12–18 years, 19–25 years, 26–35 years, and past 5 years) to examine stage-specific effects on BC risk. Analysis for daily work habits was conducted specifically among participants who self-reported having daily work or routine activities.

Statistical significance was defined as a two-sided *p*-value < 0.05. All analyses were conducted using SPSS version 21.0. Data integrity was high due to the structured face-to-face interview process; consequently, there were no missing values for the variables included in the final multivariable models, and all 2800 participants were included in the primary analysis.

## 3. Results

### 3.1. General Characteristics of Participants

Table 1 summarizes the general characteristics of BC cases and controls at baseline. Significant differences were observed for several menstrual and reproductive factors. Compared with controls, cases reported an earlier age at menarche, a later age at first full-term pregnancy, a later onset of menopause, and a higher proportion of women who had never breastfed (*p* < 0.001). Socioeconomic and lifestyle characteristics also varied. Cases reported a higher wealth score but lower levels of education, employment, energy intake, and shorter durations of moderate physical activity (*p* < 0.001). Furthermore, a family history of cancer, particularly BC, was more frequently reported among cases, and the use of oral contraceptives was also more common among cases (*p* < 0.001).

### 3.2. Physical Activity, Sedentary Behavior, and BC Risk

Table 2 presents the associations between sedentary behavior, physical activity, and BC risk. After adjusting for potential confounders, sedentary behavior (≥4 h/day) was not significantly associated with BC risk (aOR = 1.09; 95% CI: 0.91–1.30; *p* = 0.338).

Conversely, moderate physical activity (MPA) showed a significant inverse association with BC risk. Women in the highest quartile of MPA (≥1380 min/week) had an aOR = 0.37 (95% CI: 0.29–0.47; *p*-trend < 0.001) compared to the lowest quartile. Further, self-reported physical activity levels were significantly associated with BC odds. Compared to sedentary individuals, those who were slightly active (aOR = 0.51), moderately active (aOR = 0.37; 95% CI: 0.26–0.54), and highly active (aOR = 0.51; 95% CI: 0.34–0.76) were associated with lower odds of BC (*p*-trend = 0.009).

High-intensity physical activity during specific life stages revealed varied associations. No significant associations were found for high-intensity physical activity during childhood (6–11 years) or adolescence (12–18 years). However, high-intensity physical activity during ages 19–25 years (aOR = 0.65; 95% CI: 0.54–0.77; *p* < 0.001), 26–35 years (aOR = 0.65; 95% CI: 0.55–0.77; *p* < 0.001), and within the past 5 years (aOR = 0.73; 95% CI: 0.61–0.88; *p* = 0.001) were all significantly associated with lower odds of BC.

### 3.3. Daily Work Habits or Routine Activities and BC Risk

Table 3 presents the associations between daily work habits and BC risk. Frequent lifting of heavy objects showed a trend towards higher odds (aOR = 1.38; 95% CI: 0.96–1.99; *p*-trend = 0.058). Perceived fatigue after work and sweating during work tasks were both significantly associated with BC. Compared to the never/rarely category, frequent fatigue (ORa = 2.58; 95% CI: 1.52–4.36) and frequent sweating (aOR = 2.89; 95% CI: 1.79–4.67) were observed.

In contrast, walking speed for daily activities showed a significant inverse association; compared to walking slowly, walking briskly had an aOR of 0.49 (*p*-trend < 0.001). Daily walking duration showed a significant association (*p*-trend < 0.001 with aORs of 0.45 (16–30 min) and 0.44 (31–60 min). Finally, regular stair climbing was associated with BC (aOR = 0.74; *p* = 0.001), with a significant *p*-trend across frequency categories. Furthermore, a significant dose–response relationship was observed for stair climbing frequency (*p*-trend < 0.001). A significant association was observed for those climbing stairs three to four times daily (aOR = 0.49; 95% CI: 0.32–0.75), as detailed in Table 3.

## 4. Discussion

The present multicenter case–control study conducted among Moroccan women demonstrates that physical activity (PA) is significantly associated with a reduced risk of breast cancer. Our findings indicate a consistent inverse association between total Moderate-to-Vigorous Physical Activity (MVPA) and breast cancer risk. Specifically, higher levels of activity in early adulthood, brisk walking, and regular stair climbing were identified as key factors associated with reduced odds of the disease. Conversely, occupational fatigue was associated with an increased risk profile. These results suggest that promoting specific types of physical movement throughout the life course could serve as a non-pharmacological strategy for cancer prevention in this population.

In light of these results, our findings align with several international cohort studies and meta-analyses [17,18,28,29,30,31]. The observed inverse association between higher MVPA levels and BC risk aligns with evidence from several cohort studies and meta-analyses, which report similar dose–response relationships [17,28,29,30]. Regarding sedentary behavior, our results are in line with meta-analyses that reported no significant association [32], although some literature suggests variations related to occupational sitting [33]. These discrepancies likely arise from differences in measurement methods or population characteristics. Furthermore, the associations observed for vigorous activity in early adulthood, recent activity, brisk walking, and stair climbing are supported by research highlighting the importance of specific windows of exposure and the benefits of accessible activities [16,34,35,36,37,38,39,40,41,42]. Specifically, recent evidence from Raisi et al. (2024) reinforces that incidental physical activities, such as regular stair climbing, are significantly associated with a reduced risk of major chronic diseases [34]. Finally, the link identified between frequent occupational fatigue and increased BC risk, while novel in this context, is consistent with established theories regarding the role of chronic stress and systemic inflammation [43,44,45,46]. Nevertheless, it is essential to acknowledge that some findings in the literature remain heterogeneous; for instance, a study focusing on sweating during recreational exercise reported contrasting effects [47].

Regarding the biological mechanisms involved, several interrelated pathways may explain the observed associations. Regular moderate-to-vigorous physical activity is linked to reduced levels of circulating estrogens and androgens, partly through decreases in body fat and increases in sex hormone-binding globulin (SHBG), which limits bio-active estrogen availability [43,47,48,49,50,51,52,53]. Additionally, physical activity is associated with improved insulin sensitivity and lower levels of insulin-like growth factor-1 (IGF-1), which are factors involved in cell proliferation. It also correlates with anti-inflammatory effects and enhanced immune surveillance [54]. as sleep and rest are critical for maintaining immune homeostasis [44,45].

In contrast, the link identified between frequent occupational fatigue and BC risk suggests that chronic physical or psychological stress may influence the hypothalamic–pituitary–adrenal (HPA) axis [43,45,48,55]. Chronic stress-related pathways could potentially foster a tumor-promoting microenvironment through immune suppression or proinflammatory cytokine release [43,44]. However, these stress-related mechanisms require further confirmation in occupational contexts. Overall, these pathways are not mutually exclusive and may interact, although direct hormonal measurements in future studies would be required to strengthen these hypothesized associations.

The reliability of our findings is bolstered by several methodological strengths. This study’s primary strengths include its large multicenter sample (*n* = 2800), a high participation rate (93.8%), and the use of trained interviewers to minimize missing data. Furthermore, it is one of the few studies in the MENA region to analyze occupational strain (lifting, sweating, and fatigue) alongside recreational activity. Furthermore, it is one of the few studies in the MENA region to analyze occupational strain (lifting, sweating, and fatigue) alongside recreational activity. For the multivariate analysis, variable selection was based on a bivariate significance threshold (*p* < 0.20) and theoretical importance (e.g., age, BMI, reproductive history, and family history) to ensure full adjustment for potential confounders. Limitations include potential recall bias due to the retrospective design, although this was mitigated by using standardized instruments. Second, the use of hospital-based controls may introduce selection bias; however, we recruited controls from the same geographical areas as cases and excluded individuals with conditions related to the studied risk factors. Finally, while we adjusted for multiple confounders, residual confounding from unmeasured genetic or environmental factors remains possible. Nevertheless, the consistency of our results with known biological mechanisms supports the observed associations.

In terms of public health implications, our findings suggest that promoting accessible, low-cost activities like brisk walking and domestic stair climbing is associated with lower odds of BC in Morocco. Given the cultural context, integrating these recommendations into daily household and occupational routines may be more effective than focusing solely on structured leisure-time exercise. Additionally, addressing occupational stress and fatigue within workplace wellness initiatives could help reduce the regional burden of non-communicable diseases. To build upon these results, prospective cohort studies and randomized interventions in North African populations are required to further investigate potential causal pathways. Future research should prioritize objective measures (e.g., accelerometry) to reduce recall bias and in-corporate metabolic biomarkers to validate biological mechanisms. Additionally, stratifying results by menopausal status and genetic risk factors will further elucidate associations specific to the Moroccan population.

## 5. Conclusions

In conclusion, this study demonstrates a significant inverse association between higher levels of moderate physical activity, specifically brisk walking and stair climbing and breast cancer (BC) among Moroccan women. Conversely, indicators of occupational strain, such as frequent fatigue and sweating, were associated with higher odds of BC. These findings underscore the public health potential of promoting accessible daily activities as a prevention strategy in the North African context. However, given the retrospective nature of this study, further longitudinal and interventional research is essential to validate these associations and further elucidate the underlying biological mechanisms.

## Figures and Tables

**Table 1:** General characteristics of the participants (*n* = 2800).

	Controls (*n* = 1400)		Cases (*n* = 1400)		
	Mean	SD	Mean	SD	*p* Value
Age	50.47	11.41	50.92	11.36	0.30
Age at menarche (Years)	12.92	1.40	12.72	1.49	<0.001
Age at full-term pregnancy(years)	22.38	5.15	23.53	5.50	<0.001
Age at menopause(years)	48.88	5.07	50.24	5.06	<0.001
wealth score	12.65	1.75	13.17	1.89	<0.001
Energy Intake (Kcal/day)	2591.61	986.81	2497.94	747.54	<0.001
Moderate physical activity duration (min/week)	968.94	649.94	783.09	571.39	<0.001
Area of residence					1.000
Urban	954.00	68.1%	954.00	68.1%	
Rural	446.00	31.9%	446.00	31.9%	
Marital status					0.06
Single	256.00	18.3%	245.00	17.5%	
Married	954.00	68.1%	923.00	65.9%	
Divorced	87.00	6.2%	88.00	6.3%	
Widowed	103.00	7.4%	144.00	10.3%	
Education level					<0.001
Illiterate	661.00	47.2%	806.00	57.6%	
Elementary/Koranic school	296.00	21.1%	304.00	21.7%	
Secondary school	292.00	20.9%	221.00	15.8%	
High school/Technical or professional school	151.00	10.8%	69.00	4.9%	
Occupation					<0.001
Housewife	1031.00	73.6%	968.00	69.1%	
Employed	244.00	17.4%	166.00	11.9%	
Previously Employed	125.00	8.9%	266.00	19.0%	
Oral contraceptives					<0.001
Yes	644.00	46.0%	835.00	59.6%	
No	756.00	54.0%	565.00	40.4%	
Parity					0.90
Parous	1036.00	74.0%	1033.00	73.8%	
Nulliparous	364.00	26.0%	367.00	26.2%	
Breastfeeding					
Never Breastfed	14.00	1.4%	44.00	4.3%	<0.001
>0–<24 months	148.00	14.4%	287.00	27.9%	
≥24 months	869.00	84.3%	697.00	67.8%	
Family history of cancer					<0.001
Yes	266.00	19.0%	423.00	30.2%	
No	1134.00	81.0%	977.00	69.8%	
Family history of breast cancer					<0.001
Yes	77.00	5.5%	185.00	13.2%	
No	1323.00	94.5%	1215.00	86.8%	
Alcohol					0.41
Yes	9.00	0.7%	6.00	0.4%	
No	1361.00	99.3%	1394.00	99.6%	

*n*: Number of participants, SD: Standard Deviation and *p* value: Statistical significance (*p* < 0.05).

**Table 2:** Physical activity, Sedentary behavior and breast cancer risk (*n* = 2800).

		Ca/Co	OR Crude	aOR (95% CI)
Sedentary behavior				
	No (<4 h/day)	745/804	1(Ref)	1(Ref)
	Yes (≥4 h/day)	655/596	1.19(1.02–1.38)	1.09(0.91–1.30)
	*p* value		0.025	0.338
Moderate physical activity duration (min/week)				
	Q1 <420	512/384	1(Ref)	1(Ref)
	Q2 [420–840]	392/345	0.85(0.70–1.04)	0.80(0.65–0.99)
	Q3 [840–1380]	296/310	0.72(0.58–0.88)	0.62(0.49–0.79)
	Q4 ≥ 1380	200/361	0.42(0.33–0.52)	0.37(0.29–0.47)
	*p* trend		<0.001	<0.001
Self-reported Physical Activity Level (Past Year)				
	Sedentary	113/50	1(Ref)	1(Ref)
	Slightly active	360/320	0.50(0.35–0.72)	0.51(0.34–0.75)
	Moderately active	590/749	0.35(0.25–0.50)	0.37(0.26–0.54)
	High activity	337/281	0.53(0.37–0.77)	0.51(0.34–0.76)
	*p* trend		0.012	0.009
High-Intensity Physical Activity (6–11 years)				
	No	1174/1136	1(Ref)	1(Ref)
	Yes	215/225	0.93(0.75–1.13)	0.88(0.70–1.11)
	*p* value		0.451	0.287
High-Intensity Physical Activity (12–18 years)				
	No	928/891	1(Ref)	1(Ref)
	Yes	467/493	0.91(0.78–1.06)	0.90(0.75–1.07)
	*p* value		0.235	0.236
High-Intensity Physical Activity (19–25 years)				
	No	820/685	1(Ref)	1(Ref)
	Yes	574/703	0.68(0.59–0.79)	0.65(0.54–0.77)
	*p* value		0.001	<0.001
High-Intensity Physical Activity (26–35 years)				
	No	845/713	1(Ref)	1(Ref)
	Yes	555/687	0.68(0.59–0.79)	0.65(0.55–0.77)
	*p* value		<0.001	<0.001
High-Intensity Physical Activity (Past 5 years)				
	No	1029/965	1(Ref)	1(Ref)
	Yes	371/435	0.80(0.68–0.94)	0.73(0.61–0.88)
	*p* value		0.008	0.001

Ca/Co: Number of Cases/Number of Controls; OR: Odds Ratio; aOR: adjusted Odds Ratio (adjusted for age at menarche (Years), average daily caloric intake (Kcal/day), wealth score, educational level (illiterate, elementary/Koranic school, secondary school, high school /Technical or professional school), occupation (housewife, currently employed, previously employed), history of oral contraceptive use (Yes, No), age at first full-term pregnancy (nulliparous, <22 years, >22 years), breastfeeding duration (never breastfed, >0–<24 months, ≥24 month, nulliparous), age at menopause (Premenopausal, <50 years, >50 years), family history of breast cancer (Yes, No) and body mass index (<25 kg/m^2^, 25–29 kg/m^2^, ≥30 kg/m^2^); 95% CI: 95% Confidence Interval; Q1/Q4: Quartiles (Q1 = lowest level, Q4 = highest level); *p* value and *p* trend: Statistical significance (*p* < 0.05); Ref: Reference group.

**Table 3:** Daily work habits and breast cancer risk (*n* = 2800).

		Ca/Co	OR Crude	ORa (95% CI)
Sitting frequency during your job or work tasks *				
	Never/rarely	239/247	1(Ref)	1(Ref)
	Occasionally/sometimes	273/402	0.70(0.56–0.89)	0.76(0.58–1.00)
	Often	157/150	1.08(0.81–1.44)	1.10(0.79–1.51)
	*p* trend		0.975	0.784
Standing as part of daily work or routine activities *				
	Never/rarely	107/112	1(Ref)	1(Ref)
	Occasionally/sometimes	281/375	0.78(0.58–1.07)	0.71(0.50–0.99)
	Often	274/311	0.92(0.68–1.26)	0.88(0.62–1.24)
	*p* trend		0.937	0.898
Walking frequency during your job or work tasks *				
	Never/rarely	138/169	1(Ref)	1(Ref)
	Occasionally/sometimes	404/446	1.11(0.85–1.44)	1.03(0.77–1.37)
	Often	119/178	0.82(0.59–1.13)	0.84(0.59–1.20)
	*p* trend		0.238	0.338
Lifting heavy objects frequency during your job or work tasks *				
	Never/rarely	320/434	1(Ref)	1(Ref)
	Occasionally/sometimes	240/264	1.23(0.98–1.55)	1.18(0.92–1.53)
	Often	91/88	1.40(1.01–1.94)	1.38(0.96–1.99)
	*p* trend		0.017	0.058
Perceived fatigue frequency after Work *				
	Never/rarely	32/117	1(Ref)	1(Ref)
	Occasionally/sometimes	163/288	2.07(1.34–3.20)	1.37(0.84–2.23)
	Often	125/127	3.60(2.27–5.72)	2.58(1.52–4.36)
	*p* trend		<0.001	<0.001
Sweating frequency during your job or work tasks *				
	Never/rarely	80/260	1(Ref)	1(Ref)
	Occasionally/sometimes	174/204	2.77(2.01–3.83)	2.61(1.78–3.84)
	Often	72/79	2.96(1.97–4.45)	2.89(1.79–4.67)
	*p* trend		<0.001	<0.001
Walking Speed for Daily Activities				
	Slowly	293/219	1(Ref)	1(Ref)
	At a normal pace	973/988	0.74(0.61–0.90)	0.78(0.62–0.97)
	Briskly	127/185	0.51(0.39–0.68)	0.49(0.36–0.68)
	*p* trend		<0.001	<0.001
Daily Walking duration *				
	12 to 15 min	653/486	1(Ref)	1(Ref)
	16 to 30 min	188/299	0.47(0.38–0.58)	0.45(0.35–0.57)
	31 to 60 min	91/129	0.53(0.39–0.70)	0.44(0.32–0.61)
	1 to 2 h	78/67	0.87(0.61–1.23)	0.77(0.52–1.15)
	*p* trend		0.003	<0.001
Stairs climbing habit				
	No (Does not climb stairs regularly)	838/743	1(Ref)	1(Ref)
	Yes (Regularly climbsstairs)	562/657	0.76(0.65–0.88)	0.74(0.63–0.87)
	*p* value		<0.001	0.001
Daily stair Climbing Frequency				
	Once per day	104/77	1(Ref)	1(Ref)
	Twice per day	251/255	0.73(0.52–1.03)	0.84(0.58–1.22)
	Three times per day	96/151	0.47(0.32–0.70)	0.49(0.32–0.75)
	Four times per day	69/127	0.40(0.27–0.61)	0.43(0.27–0.68)
	More than five times per day (at least 40 min per day)	42/47	0.66(0.40–1.10)	0.73(0.42–1.29)
	*p* trend		<0.001	<0.001

Ca/Co: Number of Cases/Number of Controls; OR: Odds Ratio; aOR: adjusted Odds Ratio (Adjusted for age at menarche (Years), average daily caloric intake (Kcal/day), wealth score, educational level (illiterate, elementary/Koranic school, secondary school, high school/Technical or professional school), occupation (housewife, currently employed, previously employed), history of oral contraceptive use (Yes, No), age at first full-term pregnancy (nulliparous, <22 years, >22 years), breastfeeding duration (never breastfed, >0–<24 months, ≥24 month, nulliparous), age at menopause (Premenopausal, <50 years, >50 years), family history of breast cancer (Yes, No) and body mass index (<25 kg/m^2^, 25–29 kg/m^2^, ≥30 kg/m^2^). 95% CI: 95% Confidence Interval; *p* value and *p* trend: Statistical significance (*p* < 0.05); Ref: Reference group. *: Analysis was conducted among participants who self-reported having daily work or routine activities.

## Data Availability

The data presented in this study are available on request from the corresponding author.

## Supplementary Materials

The following supporting information can be downloaded at: https://www.mdpi.com/article/10.3390/epidemiologia7010022/s1, Table S1: Overview of variables, categorization, and scientific rationale for inclusion in the breast cancer risk model. Cited references: [5,8,9,12,15,17,18,21,24].

## Abbreviations

The following abbreviations are used in this manuscript:

PAMV	Physical Activity Moderate and Vigorous
PA	Physical Activity
BC	Breast Cancer
BMI	Body Mass Index
