# Supplementary material for: Association Between Physical Activity, Sedentary Behavior and Breast Cancer Risk Among Moroccan Women: A Multicenter Case–Control Study
Source: Epidemiologia (Basel). 2026 Feb 3;7(1):22. doi: 10.3390/epidemiologia7010022 (PMC12922063; doi:10.3390/epidemiologia7010022)
Supplement: Supplementary file 1 [file epidemiologia-07-00022-s001.zip › epidemiologia-4008224-supplementary.pdf]

Supplementary Table S1. Overview of variables, categorization, and scientific rationale for inclusion in the breast cancer risk model.

Variable	Categorization in the Model	Rationale for Inclusion (Scientific Justification)
**Age at menarche**	Continuous (years)	Early menarche is associated with longer lifetime exposure to endogenous estrogens, a well-established risk factor for BC [15, 17].
**Body Mass Index (BMI)**	<25, 25–29, ≥30 kg/m²	Adiposity, particularly in postmenopausal women, increases BC risk due to higher levels of estrogen produced by adipose tissue and chronic inflammation [15, 18].
**Family history of BC**	Yes / No	A family history reflects genetic predisposition, which is one of the strongest non-modifiable risk factors for BC [8, 12].
**Age at first full-term pregnancy**	Nulliparous, <22, ≥22 years	Early full-term pregnancy promotes early differentiation of breast cells, which is known to have a long-term protective effect against BC [9].
**Parity and Breastfeeding**	Never, >0–<24, ≥24 months	Breastfeeding reduces BC risk by delaying the re-establishment of ovulatory cycles and reducing lifetime estrogen exposure [18].
**Menopausal status / Age**	Premenopausal, <50, ≥50 years	The risk profile of BC changes significantly after menopause due to the shift from ovarian to peripheral estrogen production [17].
**Oral contraceptive use**	Ever / Never	Exogenous hormone use is associated with a slight but significant increase in BC risk during the period of use [9].
**Education and Wealth Score**	Categorical (levels)	These variables serve as proxies for Socio-Economic Status (SES), which influences lifestyle choices, diet, and access to healthcare [5].
**Daily energy intake**	Continuous (kcal/day)	High caloric intake is related to metabolic health and obesity, both of which are linked to cancer development [24].
**Occupation**	Housewife, Employed, Previously employed	Professional status can influence physical activity levels, stress, and exposure to environmental factors [21].
